# Case report: Splenic inflammatory pseudotumor-like follicular dendritic cell sarcoma (IPT-like FDCS): a trial of immunotherapy and review of the literature

**DOI:** 10.3389/fonc.2024.1360726

**Published:** 2024-06-20

**Authors:** K. A. Resnick, C. Monroe, I. Siddiqi, E. Tam

**Affiliations:** ^1^ Department of Medicine, University of Southern California (USC)/Los Angeles General, Los Angeles, CA, United States; ^2^ Department of Hematopathology, Keck Medicine of USC, Los Angeles, CA, United States; ^3^ Department of Bone Marrow Transplant, USC Norris Comprehensive Cancer Hospital, Los Angeles, CA, United States

**Keywords:** inflammatory pseudotumor-like follicular dendritic cell sarcoma, IPT-like FDCS, recurrence, immunotherapy, case report

## Abstract

Inflammatory pseudotumor-like follicular dendritic cell sarcoma (IPT-like FDCS) is a rare malignancy with fewer than 150 cases in the literature. IPT-like FDCS follows an indolent course with most cases definitively managed with surgical resection. We present a case of IPT-like FDCS with multiple recurrences with a trial of immunotherapy. The patient initially presented with splenic involvement requiring splenectomy, subsequently recurring in the liver requiring hepatic resections. Afterwards, there was recurrence with pelvic/small bowel involvement for which treatment was trialed with ipilimumab and nivolumab. The patient progressed despite dual immune checkpoint inhibitor therapy requiring a small bowel resection. To date, this is the first case of immunotherapy use in IPT-like FDCS. Therefore, more evidence is needed to support additional treatments in recurrent IPT-like FDCS after resection.

## Introduction

Follicular dendritic cell sarcoma (FDCS) was first recognized in 1986 as an uncommon neoplasm resulting from proliferation of follicular dendritic cells (1). While the majority of FDCS are localized to lymph nodes, extranodal FDCS has arisen across multiple organs including the tonsils, nasopharynx, GI tract, and retroperitoneum. The clinical course is usually indolent and managed via surgical resection. However, local recurrence is common in 40–50% of cases (2, 3). Higher risk FDCS, which are larger in size or located intra-abdominally, display more aggressive spread, which can require the use of chemo or radiation therapy (3, 4).

Inflammatory pseudotumor-like follicular dendritic cell sarcoma (IPT-like FDCS) has been recognized as a separate subtype from classic FDCS by the WHO 2022 criteria (5). It is characterized by positive markers for follicular dendritic cells including CD21 and CD35, with a histology featuring diffuse inflammatory cells (6). Unlike FDCS, IPT-like FDCS affects predominantly female patients, mostly originating intraabdominally and with associated Epstein Barr virus (EBV) positivity (7, 8). IPT-like FDCS is a rarer variant, with fewer than 150 cases documented in literature (9). It is generally understood to be more indolent than classic FDCS, with fewer than 15 cases documenting disease recurrence after resection (7–10). While some case reports have trialed the use of chemotherapy or targeted therapies (11, 12), overall knowledge and evidence on the efficacy of these interventions is severely limited.

## Case description

**Figure 1:**
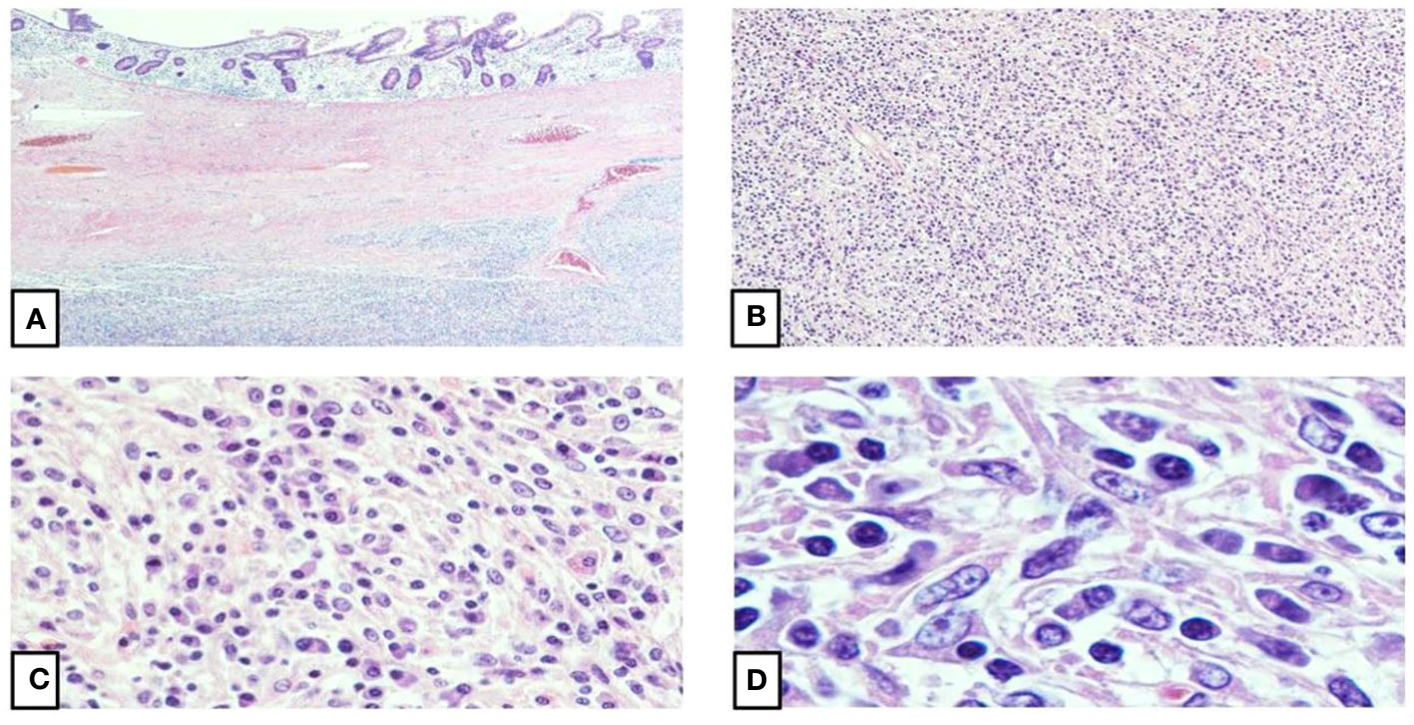
Histomorphology of inflammatory pseudotumor-like follicular dendritic cell| sarcoma. **(A)** The intestinal mucosa shows infiltration by a well-circumscribed, spindle cell proliferation (4X). **(B)** The spindle cell proliferation shows atypical spindle cells arranged in fascicles and suggested whorls with focal storiform patterned areas (10X). **(C, D)** Higher magnifications show oval to spindle cells with vesicular nuclei, stippled chromatin, small, centrally placed conspicuous nucleoli, scant to moderate amounts of eosinophilic and fibrillar cytoplasm, and mostly indistinct, syncytial borders. A small proportion of the oval to spindle cells demonstrate binucleation with occasional multinucleation and nuclear pseudoinclusions. A lymphoplasmacytic infiltrate with dispersed eosinophils and rare neutrophils constitute the background (40X, 100X).

**Figure 2:**
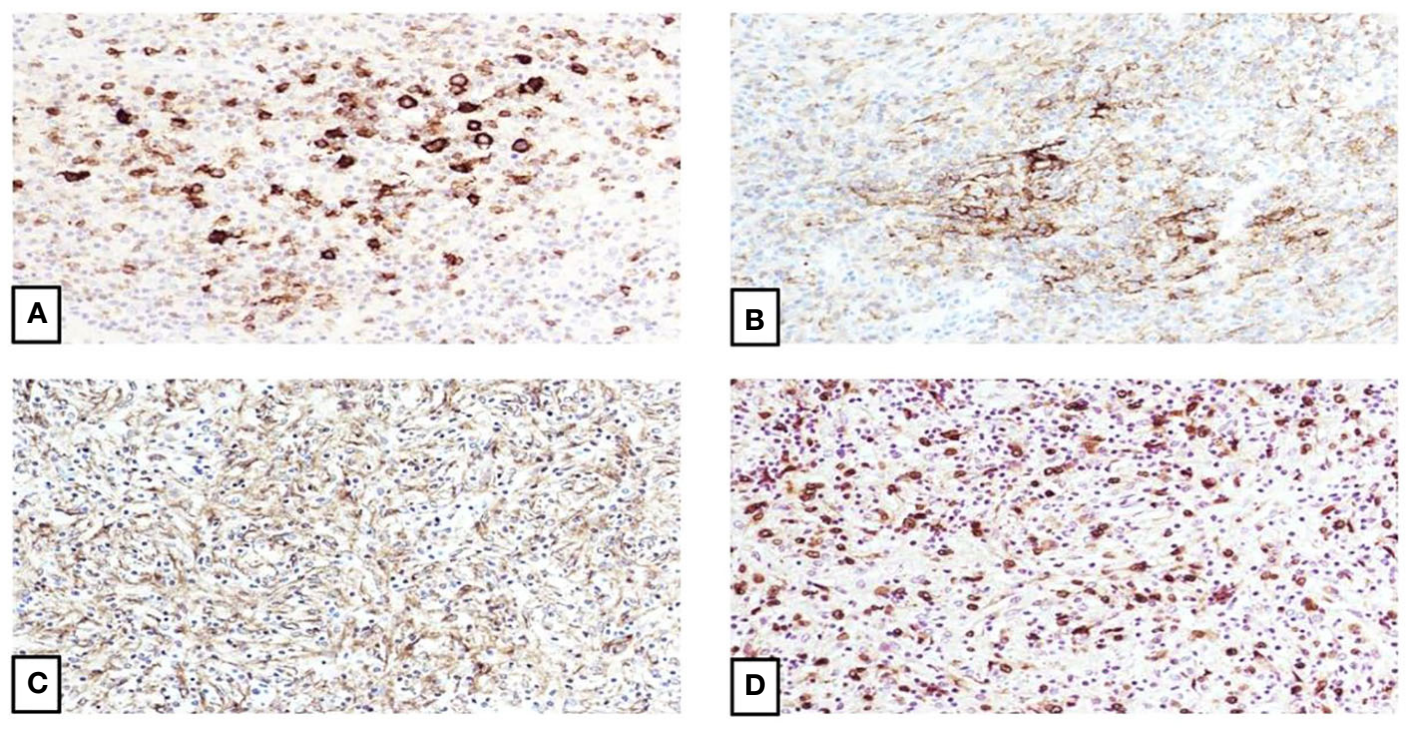
Immunophenotype of inflammatory pseudotumor-like follicular dendritic cell sarcoma. **(A)** Focal staining of the spindle cells by CD23 confirms the follicular dendritic cell differentiation (20X). **(B)** CD35 also shows focally strong staining (20X). **(C)** EGFR is also positive (20X). **(D)** EBV staining is strongly and diffuse positive, which correlates with the proposed theory of tumor derivation from EBV-infected mesenchymal cells and subsequent follicular dendritic cell pathway differentiation (20X).

The patient was a 22-year-old female who initially presented in 2016 for abdominal pain. She was otherwise healthy, nulliparous, with no significant past medical history or family history. On CT scans, she was found to have a 12.4 cm splenic mass with rupture requiring splenectomy. Splenic pathology demonstrated an EBV positive stromal proliferation with protein deposition and recent hemorrhage consistent with IPT-like FDCS. Immunohistochemical staining was positive for CD23 and CD35 with a Ki67 proliferative index of 20%, p53 (20% of tumor cells with overexpression) and EBER positivity. Immunohistochemical staining can be seen in **Figures 1**, **2**. Resected margins were clear on biopsy and patient followed up with an outside network. In January 2021, she began developing abdominal pain again and an abdominal MRI demonstrated a new enhancing liver lesion initially 2.7 cm in size. Over the course of 11 months, repeated scans documented the growth of the primary right hepatic lesion to 9.6 cm with two additional smaller lesions in separate hepatic segments. The patient underwent CT guided core biopsy of the largest lesion. Biopsy pathology was reviewed multiple times prior to being determined as consistent with her prior IPT-like FDCS. PET CT performed in December 2021 confirmed three hypermetabolic liver lesions, no focal uptake at the site of her prior splenectomy, and no additional foci of hypermetabolic activity. She underwent a multi-segment hepatic resection in February 2022, the largest lesion at time of resection measuring 11cm. Intraoperatively, there was no extrahepatic disease noted and all three lesions were removed in entirety. Hepatic pathology confirmed disease recurrence.

Surveillance PET scan was performed in July 2022, which demonstrated no further liver disease, but a new hypermetabolic right-sided pelvic mass. Further CT abdominal imaging confirmed a 7.5 cm soft tissue mass of the right pelvis and side wall with a 3.3 cm right adnexal cystic lesion. CT guided biopsy in August 2022 demonstrated inflammatory EBV-positive IPT-like FDCS recurrence and interval imaging in September 2022 showed increased size of the mass, now abutting the small bowel and the colon with no invasion.

The patient’s case was discussed in tumor board given limited evidence regarding treatment options for recurrent IPT-like FDCS. Based on limited reports of targeted therapies for FDCS, additional testing was sent for hormonal and checkpoint targets. Estrogen (ER), progesterone (PR), and HER-2 testing was negative for the liver and pelvic masses. Immunohistochemical PD-L1 testing demonstrated 1–5% staining of the lesional dendritic cells from the liver and pelvic masses. Additional testing from the hepatic tumor using the Tempus xT assay demonstrated a tumor mutation burden score of 4.2 mutations/Mb (55^th^ percentile, microsatellite stable). Based on these results and the relative indolence of disease, the decision was made to start a trial of immunotherapy prior to more toxic systemic chemotherapy regimens.

**Figure 3:**
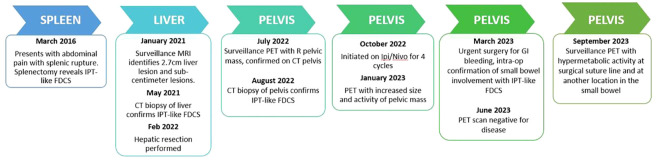
Clinical timeline demonstrating the location and pattern of tumor recurrence.

**Figure 4:**
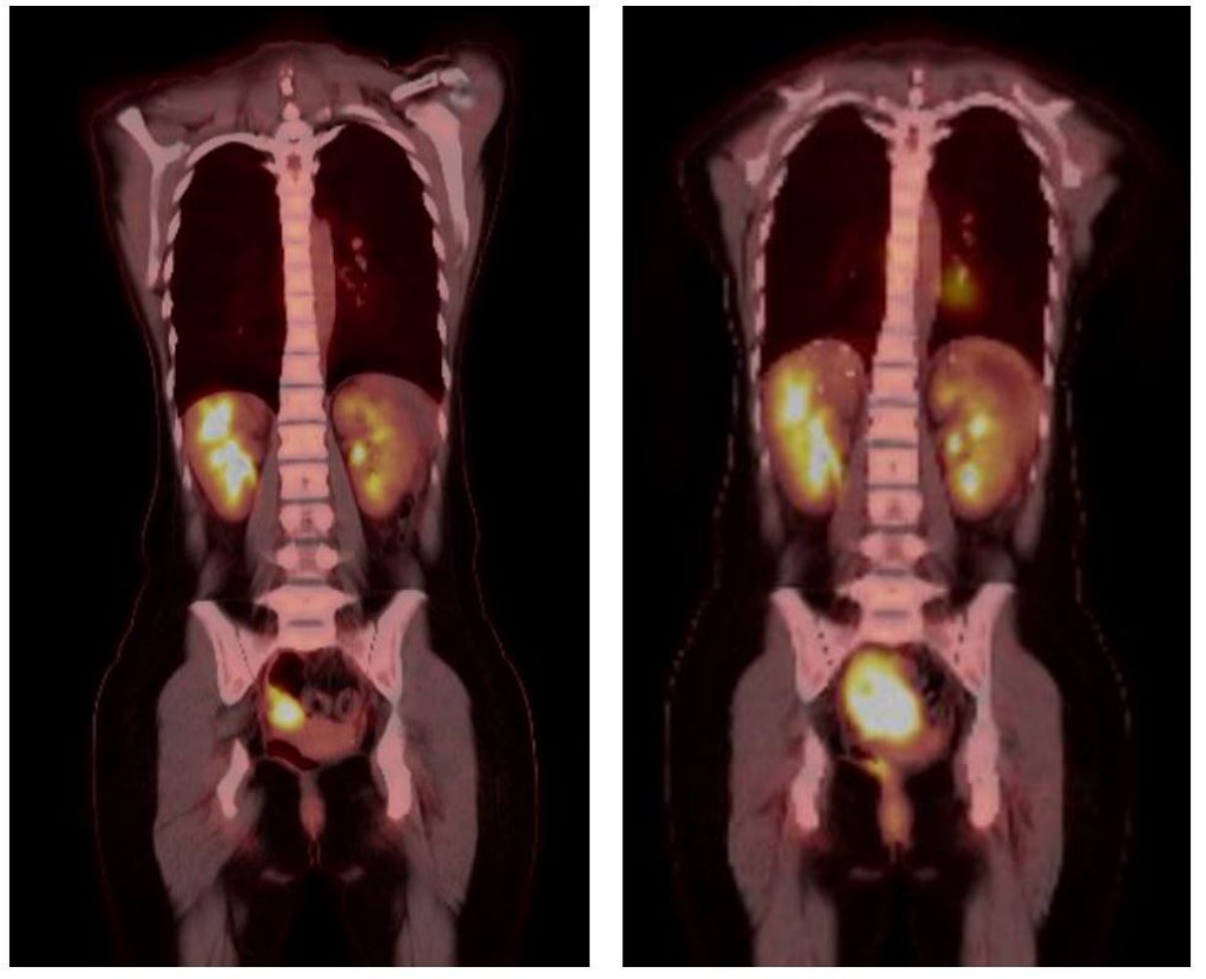
PET scans performed prior (left) to dual immune checkpoint inhibitor use and after (right) four cycles of combination ipilimumab/nivolumab. Scans demonstrate increased size and avidity of the pelvic small bowel mass after treatment.

The patient received four cycles of combination ipilimumab 1mg/kg with nivolumab 3mg/kg, which were tolerated without issues. The timeline of the full clinical course can be seen in **Figure 3**. Repeat MRI and PET scans in January 2023 showed increased size of the right pelvic mass to 7.8 x 5.5 x 5.5cm consistent with progression, but possible pseudo-progression. By March 2023, new enlarging presacral lymph nodes and an external iliac lymph node were noted in additional to interval growth, concerning for progression of disease. The PET scans before and after immune checkpoint inhibitor therapy can be seen in **Figure 4**. Clinical course was complicated by acute gastrointestinal bleeding requiring urgent resection. Intraoperatively, the pelvic mass appeared to involve the small bowel, posteriorly a portion of the rectosigmoid colon, and the peritoneum surrounding the right ovary and fallopian tube. Resections of these organs were performed. Pathologic review demonstrated limited involvement of a segment of small bowel with morphological and immunophenotypic patterns consistent with IPT-like FDCS as seen in the 2016 and 2022 resections. The right ovary demonstrated a spindle cell lesion. Resected margins and remaining organs were negative for the IPT-like FDCS. Anastomoses were created during the procedure after resection of the small bowel and rectosigmoid colons. The patient has since undergone repeat PET scans in June and December of 2023, which demonstrate no specific evidence of recurrence or metastasis.

## Discussion

Inflammatory pseudotumor-like follicular dendritic cell sarcoma (IPT-like FDCS) is a rare and usually indolent disease. Since its first recognition in 2001 (7), case reports in the literature have been limited to fewer than 150 cases (9). Of those cases, the majority were definitively managed with surgical resection alone, which is considered curative for many cases. The indolent nature of this disease is often a defining feature compared to classic FDCS (11). There are less than 15 documented cases of IPT-like FDCS recurrence after resection (9), therefore little evidence supports any further treatments beyond resection.

The use of chemotherapy or targeted therapy for more aggressive IPT-like FDCS has been documented in two case reports (11, 12). These therapies were trialed based on management of the more aggressive variant FDCS or used for ongoing symptom control. In a series of six patients with IPT-like FDCS, one patient experienced relapse to the liver and an aortocaval lymph node after initial hepatic lobectomy. They were subsequently treated with CHOP and documented to have no further recurrences after 7 years of follow-up (12). Another case of IPT-like FDCS with associated paraneoplastic pemphigus and myasthenia gravis was managed with rituximab for refractory myasthenia symptoms. Although the rituximab was initially successful, the patient did not undergo any further definitive management of her IPT-like FDCS and had a recurrence of her paraneoplastic pemphigus (11).

Given the limited data on systemic therapies for IPT-like FDCS, we reviewed management of FDCS, where multiple approaches including total resection, chemotherapy, radiotherapy, and immunotherapy have been trialed. One review of 66 FDCS cases found that patients experience heterogenous outcomes depending on the extent of disease and treatments used. Extra-nodal, bulky, or intraabdominal disease was associated with worse prognosis. Treatment with total resection improved survival and treatment with consolidative radiotherapy reduced locoregional recurrence. Patients with widespread disease received systemic chemotherapy comprised of gemcitabine and a taxane with good response (13), but most ultimately relapse. Other cases have noted the similarity of FDCS to malignant lymphoma and soft tissue sarcoma and used chemotherapy regimens with doxorubicin, ifosfamide, and CHOP (14) successfully.

More recent cases have explored the use of immunotherapies in the management of FDCS. Immunohistochemical staining of PD-L1 has been reported positive in 50–80% of FDCS, leading some to question whether immune checkpoint inhibitors (ICI) may be effective. The use of ICI has so far yielded mixed results (15–17). In one case of metastatic FDCS involving the mediastinum and liver, the authors trialed salvage nivolumab due to poor tolerance of CHOP. Immunohistochemical staining for PD-L1 was not included and the patient ended up progressing rapidly through salvage nivolumab (16). Another series of two FDCS cases treated with combination ipilimumab/nivolumab reported better outcomes. Both patients in the series had already undergone initial surgical resection with intra-abdominal recurrence. PD-L1 testing demonstrated combined tumor scores of 60–70% and 10% respectively. One patient underwent TMB testing resulting in TMB low (17). Both patients experienced radiographic response after 8–12 weeks, which was promising, despite the short follow up course.

In our IPT-like FDCS patient’s case, PD-L1 positivity and tumor mutation burden (TMB) was noted during multidisciplinary discussion. Given the generally indolent nature of the disease, limited evidence for systemic therapy, and desire to avoid more toxic regimens, the decision was made to pursue combination ipilimumab/nivolumab immunotherapy over chemotherapy. Our patient received four cycles with poor radiographic response and evidence of progression on both follow up MRI and PET.

Some possible contributing factors to our patient’s poor response to combination immune checkpoint inhibitor therapy may include the degree of PD-L1 positivity and TMB, and the unique histopathology of IPT-FDCS. Both PD-L1 and TMB have been used as biomarkers to predict tumor response to immune checkpoint inhibitor therapy (18, 19). Comparatively, our patient’s PD-L1 positivity of 1–5% was lower than the levels noted in FDCS case reports (17), which may contribute to reduced response (18). Different cutoffs for TMB across tumor types have been used to predict responsiveness to immune checkpoint inhibitors and improved survival following treatment (19). However, it is unclear if higher TMB would have affected outcomes. An additional consideration includes the pathological differences between IPT-like FDCS and FDCS. The anti-tumor efficacy of ICIs has increasingly been linked with the tumor microenvironment and the ability to upregulate an immune response against malignant cells (20). Some authors have speculated that the pathology of FDCS as a proliferation of antigen-presenting-cells and high expression of PD-L1 may heighten the immunogenic effect of ICIs (17). It is possible that the unique histology of IPT-like FDCS with its pre-existing background of inflammatory cells may not mount the same immune response.

To our knowledge, this is the first case of immune checkpoint inhibitor use in IPT-like FDCS. Our case demonstrates limitations in the applicability of ICIs for IPT-like FDCS. Additionally, our case describes the unique course of IPT-like FDCS with multiple recurrences, a progression that is rarely seen within the disease. This case adds to existing literature to advance the management of recurrent IPT-like FDCS and further research is needed to continue to advance patient outcomes.

## Patient perspective

The patient provided written consent for participation in this case report. Decisions to pursue a trial of immunotherapy were based on joint discussions regarding her preference to avoid chemotherapy and hysterectomy for future childbearing. She has tolerated her treatments well with a stable BMI, participated in acute rehab post-operatively and currently leads an active lifestyle.

## Data availability statement

The original contributions presented in the study are included in the article/supplementary material. Further inquiries can be directed to the corresponding author/s.

## Ethics statement

Written informed consent was obtained from the individual(s) for the publication of any potentially identifiable images or data included in this article.

## Author contributions

KR: Writing – review & editing, Writing – original draft. CM: Writing – review & editing, Writing – original draft, Data curation. IS: Writing – review & editing, Writing – original draft. ET: Writing – review & editing, Writing – original draft.
